# Panallergen sensitization in children with eosinophilic esophagitis: a pilot study

**DOI:** 10.3389/falgy.2026.1806340

**Published:** 2026-06-24

**Authors:** Raffaella De Santis, Valentina Silvera, Cristina Ferrigno, Laura Gianolio, Cecilia Mantegazza, Cristina Cocuccio, Lorenzo Norsa, Miriam Acunzo, Gian Vincenzo Zuccotti, Elena Pozzi, Enza D'Auria

**Affiliations:** 1 Department of Paediatrics, Buzzi Children’s Hospital, University of Milan, Milan, Italy; 2 Department of Biomedical and Clinical Sciences, University of Milan, Milan, Italy

**Keywords:** eosinophilic esophagitis, panallergen sensitization, food sensitization, children, proton pump inhibitor response

## Abstract

**Background:**

Eosinophilic esophagitis (EoE) is a chronic, immune-mediated disorder associated with atopic comorbidities and may be considered a late manifestation of the atopic march. This study aimed to evaluate the prevalence of atopic comorbidities, sensitization to food allergens and panallergens in pediatric EoE patients, and to explore the potential association between panallergen sensitization and response to treatment.

**Methods:**

We conducted a single-center observational study on pediatric EoE patients, followed at Vittore Buzzi Children's Hospital in Milan. Clinical, endoscopic, and histological data, together with sensitization profiles to food allergens and panallergens [profilins, PR-10 proteins, and lipid transfer proteins (LTPs)] were collected. Patients were classified into two groups according to their response to PPI therapy. *Group 1* included patients who maintained histological remission on PPIs. *Group 2* included patients who either failed to respond to PPI induction therapy or relapsed on maintenance dosing, thus requiring a therapeutic switch.

**Results:**

Twenty-four patients (median age 11.5 years, IQR 9–13; 23 males) were enrolled. 79% (19/24) had a family history of atopy and 19/24 (79%) had allergic comorbidities. Sensitization to food allergens was observed in 17/24 (71%); panallergen sensitization was detected in 15/24 patients (63%). *Group 2* showed a significantly higher prevalence of panallergen sensitization (78% vs. 17%, *p* = 0.015) and atopic dermatitis (56% vs. 17%, *p* < 0.05) compared to *Group 1*. Sensitization to profilins and LTPs was associated with lack of PPI response.

**Conclusions:**

This study is the first to address panallergen sensitization in a pediatric cohort of EoE patients. Our findings suggest that panallergen sensitization might be associated with response to PPI. However, these preliminary findings require confirmation in larger, powered studies.

## Highlights

Panallergen sensitization, together with a personal history of atopic dermatitis in early childhood, may be associated with a phenotype of EoE characterized by lower PPI responsiveness. Further studies are needed to clarify the potential clinical relevance of these findings.

## Introduction

Eosinophilic esophagitis (EoE) is a chronic, immune-mediated disorder characterized by genetic predisposition, epithelial barrier dysfunction, and type 2 (Th2) inflammation (1).

Clinically, it presents with symptoms of esophageal dysfunction and it is histologically defined by eosinophilic infiltration of the esophageal mucosa (≥15 eosinophils per high-power field, HPF) (1, 2).

Atopic conditions, including asthma, allergic rhinitis, atopic dermatitis, and IgE-mediated food allergies, are more prevalent in EoE patients than in the general population, suggesting that this disease may represent a late manifestation of the “atopic march” (3). *Food allergens* play a key role in the pathogenesis of EoE, as demonstrated by the efficacy of elemental and elimination diets in EoE (4–6).

Recent studies have highlighted the emerging role of panallergens in EoE (7, 8). These highly conserved proteins, which share epitopes between pollens and plant-based foods like fruits and vegetables, promote significant cross-reactivity and multiple sensitizations. Key panallergen families include profilins (e.g., rPhl p12 from grasses), PR-10 proteins (e.g., rBet v1, the primary birch pollen allergen), and lipid transfer proteins (LTPs, such as rTri a 14 from wheat).

Currently, the management of pediatric EoE includes three main first-line options for induction of remission: proton pump inhibitors (PPIs), topical corticosteroids (TCS), and food elimination diets (FED). The choice of treatment depends on the patient's age, symptom severity, and individual preferences (1). It is known that double-dose PPI therapy induces clinical and histological remission in approximately half of EoE patients, both in children and adults. Up to 45% of initial responders subsequently relapse on maintenance dosing; sustained histological remission with PPI monotherapy is ultimately achieved in only approximately one-quarter to one-third of all EoE patients (9). To date, no reliable clinical, laboratory, or histological criteria have been identified to determine who may best be treated using a particular therapy.

While panallergen sensitization is well-documented in adult EoE, with studies reporting a prevalence of up to 70% (10–13), limited data exist on its prevalence in pediatric EoE (14, 15).

This study aims to determine the prevalence of sensitization to food allergens and panallergens in a pediatric EoE cohort and to explore the potential role of panallergen sensitization.

## Methods

A single-center observational study was conducted at Vittore Buzzi Children's Hospital in Milan. This retrospective-prospective study included 24 patients with a diagnosis of EoE, admitted and followed at our multidisciplinary gastro-allergy eosinophilic gastrointestinal diseases service.

This study was conducted in accordance with the Helsinki Declaration and its later amendments. The study was approved by the local Ethics Committee, and informed consent was obtained from the parents or legal guardians of all participating children.

PPIs were proposed as first-line induction therapy at 1–2 mg/kg/day for at least 8 weeks in all patients. In those achieving clinical and histological response, PPI therapy was continued at half the induction dose as maintenance treatment. Patients who failed to respond to PPI induction therapy or relapsed on PPI maintenance treatment were switched to alternative treatments, including topical corticosteroids, dietary therapy, or Dupilumab in accordance with current guidelines (1, 16).

Demographic and clinical data were collected, including family history of atopy, atopic comorbidities, and therapeutic history from diagnosis onward. Clinical, endoscopic, and histological data were obtained at diagnosis and during follow-up. Baseline disease characteristics were described using clinical features, the Eosinophilic Esophagitis Endoscopic Reference Score (EREFS), and peak eosinophil count (PEC) at diagnosis, when available.

Additionally, a standardized allergy panel test, including total and specific IgE levels for major food allergens along with panallergens, was performed at the diagnosis of EoE or as early as possible by using ImmunoCAP (Thermo Fisher Scientific), with a cutoff value of ≥0.35 kUA/L for positivity.

Panallergens included profilins (rPhl p 12, rBet v 2), PR-10 proteins (rBet v 1, rAra h 8, rCor a 1, rMal d 1, rPru p 1), and lipid transfer proteins (LTPs) (rTri a 14, rAra h 9, rCora 8, rJug r 3, rPru p 3).

Endoscopic reassessment was performed in all patients after PPI induction (approximately 8 weeks). Symptom improvement was recorded as part of the clinical evaluation but was not used as a primary criterion for group classification.

Patients were classified into two groups according to their response to PPI therapy. *Group 1* included patients who maintained histological remission on PPIs. *Group 2* included patients who either failed to respond to PPI induction therapy or relapsed on maintenance dosing, thus requiring a therapeutic switch.

Continuous variables with non-normal distribution were expressed as median and interquartile range (IQR) and compared using the Mann–Whitney *U* test. Categorical variables were presented as frequencies and percentages and analyzed using Fisher's exact test. A *p*-value <0.05 was considered statistically significant. Odds ratios (ORs) with 95% confidence intervals (CIs) were calculated for selected categorical variables to provide a descriptive measure of association. All statistical analyses were performed using STATA version 12.0 (StataCorp).

## Results

### Population baseline characteristics

The study included 24 pediatric patients diagnosed with EoE, with a median age of 11.5 years (IQR 9–13) and a predominance of males (23/24, 96%). The median frame-time from diagnosis to data collection point was 2.2 years (IQR 1–4.2). A family history of atopy was reported in 19/24 patients (79%), and atopic comorbidities were common: 12/24 (50%) had allergic rhinitis, followed by atopic dermatitis (10/24, 42%), asthma (8/24, 33%), and confirmed IgE-mediated food allergies (6/24, 25%). Common symptoms at disease onset included dysphagia (20/24, 83%) and food impaction (18/24, 75%). Demographic and clinical characteristics are summarized in Table 1.

**Table 1:** Overview of demographic and clinical characteristics in the study cohort.

Characteristic	Total cohort (*N* = 24)
Male sex	23 (96%)
Median age at diagnosis [years, median (IQR)]	11.5 (9–13)
Median age at symptom onset [years, median (IQR)]	8 (6–10.25)
Median diagnostic delay [years, median (IQR)]	2.5 (1–3)
Median follow-up [years, median (IQR)]	2.2 (1–4.2)
Atopic comorbidities	
Any	19 (79%)
Asthma	8 (33%)
Allergic rhinitis	12 (50%)
Atopic dermatitis	10 (42%)
IgE-mediated food allergy	6 (25%)
Family history of atopy	19 (79%)
Symptoms at onset	
GERD-like symptoms	5 (21%)
Dysphagia	20 (83%)
Food impaction	18 (75%)
Vomiting	7 (29%)
Abdominal pain	2 (8%)
Failure to thrive	5 (21%)

GERD, gastroesophageal reflux disease.

Nineteen out of 24 (79%) patients had elevated total IgE and most patients (17/24, 71%) were sensitized to multiple food allergens. Wheat was the most common food allergen (19/24, 79%), followed by nuts (16/24, 67%), sesame (15/24, 63%), fruits (15/24, 63%), milk (14/24, 58%), soy (10/24, 42%), shrimp (10/24, 42%) and eggs (6/24, 25%). Sensitization to meat (5/24, 21%) and fish (3/24, 13%) was less common. Notably, 17/24 (71%) were polysensitized to three or more food allergens.

**Figure 1:**
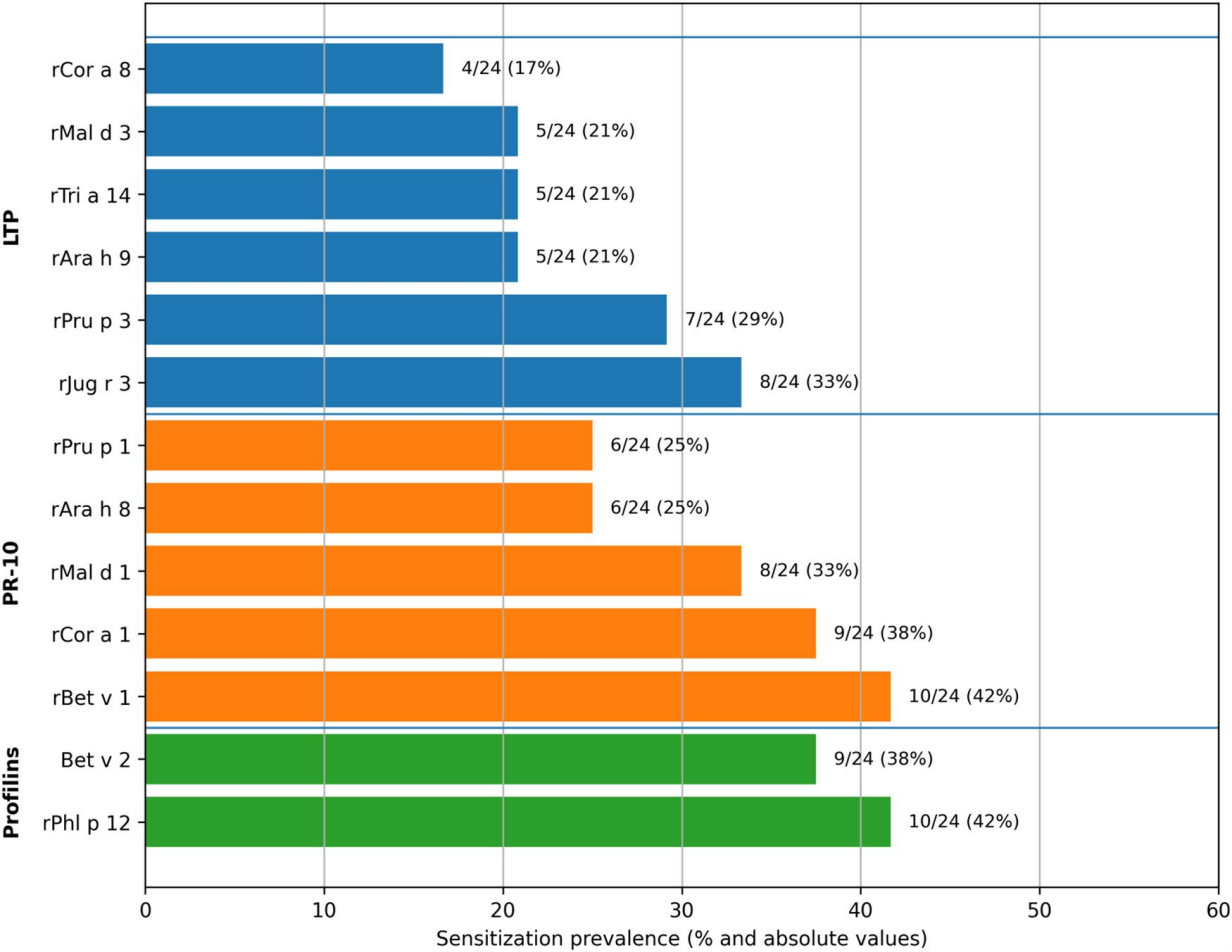
Panallergen sensitization profile in all EoE patients (*n* = 24).

Sensitization to panallergens was found in 15/24 patients (63%), with at least one class involved. With regard to profilins, 10/24 (42%) were sensitized to rPhl p 12 and 9/24 (38%) to Bet v 2. Regarding PR-10 proteins, 10/24 (42%) tested positive for Bet v 1 and 9/24 (38%) for rCor a 1. Among LTPs, 8/24 (33%) had IgE for rJug r 3 while IgE to rPru p 3 was detected in 7/24 (29%) of cases, as shown in Figure 1.

### Comparison between Group 1 and Group 2

Six out of 24 patients (25%) maintained histological remission on PPIs throughout follow-up (*Group 1*), while 18/24 (75%) failed to maintain remission and required a treatment modification, switching to topical corticosteroids (16/18, 89%) or Dupilumab (2/18, 11%) (*Group 2*).

The median age at symptom onset was higher in *Group 1* compared to *Group 2* (9.50 years; IQR 8–12 vs. 8 years; IQR 6–10) although the difference was not statistically significant. Median follow-up was comparable between groups (2.8 years [IQR 1.9–4.8] in *Group 1* and 4.2 years [IQR 2.7–4.7] in *Group 2*)*.* Atopic dermatitis was more common in *Group 2*, affecting 10/18 patients (56%) compared to 1/6 (17%) in *Group 1* (*p* < 0.05; OR 6.25, 95% CI 0.6–64). Baseline clinical features, including dysphagia-related symptoms and food impaction at presentation, were comparable between groups. PEC at diagnosis was also similar between *Group 1* and *Group 2* (46 [IQR 40–52] vs. 45 [IQR 28–55]). In contrast, EREFS at diagnosis was higher in *Group 2* than in *Group 1* (4 [IQR 3–5] vs. 2 [IQR 2–2], *p* = 0.01).

**Figure 2:**
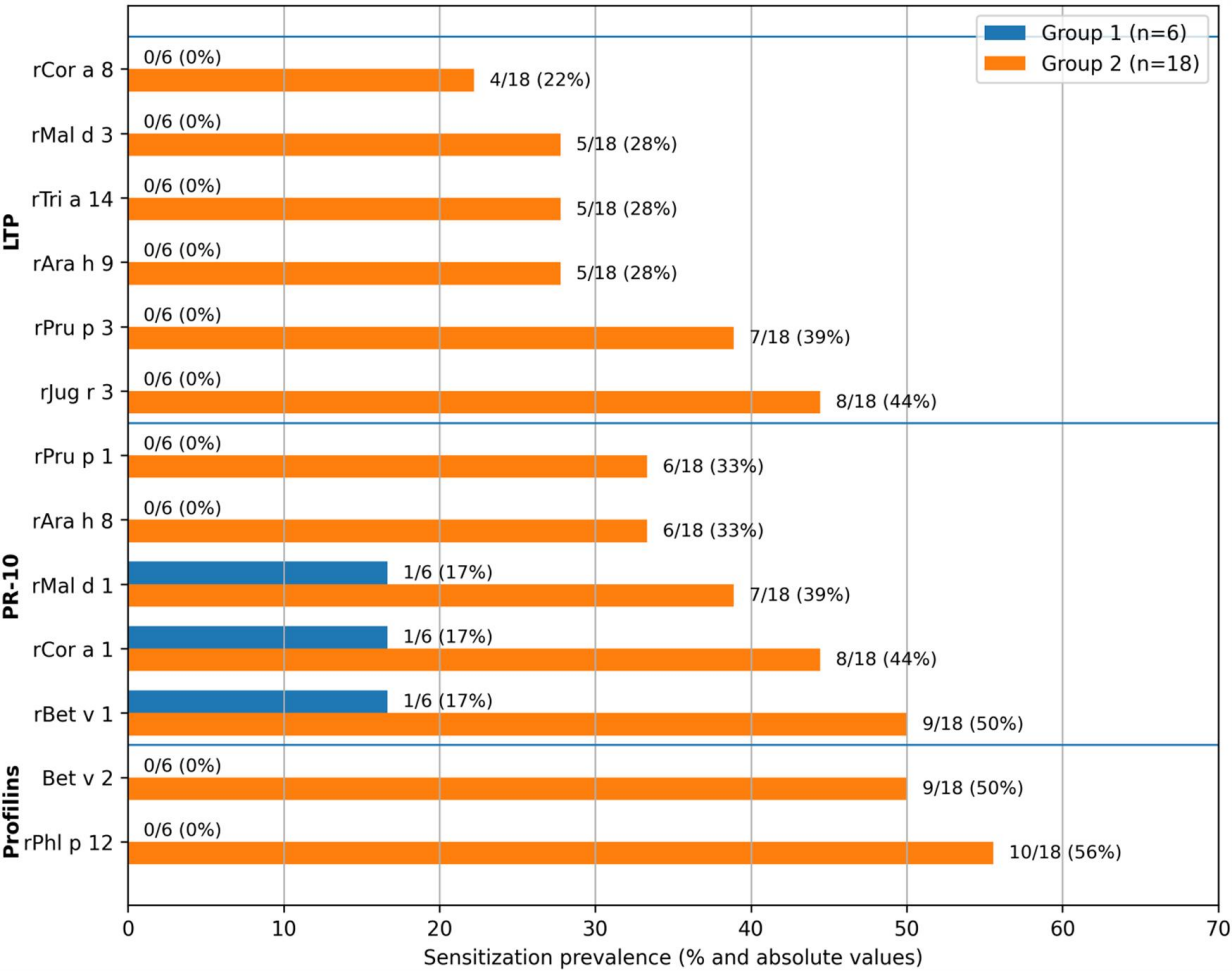
Panallergen sensitization profiles in Group 1 and Group 2.

Moreover, panallergen sensitization was more frequent in *Group 2*, with 14/18 patients (78%) sensitized to at least one panallergen class compared to 1/6 (17%) in *Group 1* (*p* = 0.015; OR 17.5, 95% CI 1.6–190). Specifically, sensitization to profilins and LTPs was notably higher in *Group 2*, as illustrated in Figure 2.

All analyzed variables, including those without statistically significant differences, are reported in Table 2*.*

**Table 2:** Comparison of demographic, clinical, and allergic sensitization patterns between Group 1 and Group 2.

Variables	Group 1	Group 2	*p*-value
Male sex	6/6 (100%)	17/18 (94%)	1
Median age at diagnosis (years, median, IQR)	12 (10–15)	11.5 (9–12)	0.28
Median age at symptom onset (years, median, IQR)	9.5 (8–12)	8 (6–10)	0.14
Family history of atopy	5/6 (84%)	14/18 (78%)	1
Any atopic comorbidity	3/6 (50%)	16/18 (89%)	0.08
Asthma	3/6 (50%)	5/18 (28%)	0.36
Allergic rhinitis	2/6 (33%)	10/18 (56%)	0.64
IgE-mediated food allergy	1/6 (17%)	5/18 (28%)	1
Atopic dermatitis	**1/6 (17%)**	**10/18 (56%)**	**<0.05**
Food impaction at presentation	5/6 (83%)	13/18 (72%)	1
Dysphagia/ odynophagia/ solid food refusal at presentation	5/6 (83%)	15/18 (83%)	1
EREFS at diagnosis (median, IQR)	**2 (2–2)**	**4 (3–5)**	**0.01**
Peak eosinophil count at diagnosis	45.5 (40–52)	45 (28–55)	1
Food sensitization	5/6 (83%)	15/18 (83%)	1
Panallergen sensitization	**1/6 (17%)**	**14/18 (78%)**	**0.015**
Profilin sensitization	**0/6 (0%)**	**10/18 (56%)**	**<0.05**
r Phl p12 Profilin sensitization	**0/6 (0%)**	**9/18 (50%)**	**<0.05**
LTP sensitization	**0/6 (0%)**	**10/18 (56%)**	**<0.05**
PR-10 sensitization	1/6 (16.7%)	9/18 (50.0%)	0.34

Bold values indicate statistically significant *p*-values.

IQR, interquartile range.

## Discussion

This study provides new insights into the sensitization profile of children with EoE, emphasizing the high prevalence of sensitization not only to food allergens but also to panallergens, an area still poorly characterized in this population.

In agreement with the available literature (17), our cohort showed a strong atopic background, with over 75% of patients reporting a family history of atopy and a high prevalence of atopic comorbid conditions such as allergic rhinitis, atopic dermatitis, and asthma. These findings reinforce the hypothesis that EoE may represent the final step of the atopic march (3).

In our cohort, sensitization to foods was common, with wheat as the most frequent food allergen (79%), followed by nuts (67%), sesame (63%), fruits (63%), and milk (58%).

Notably, more than 70% of patients were polysensitized to three or more food allergens.

The high prevalence of wheat sensitization may reflect cross-reactivity phenomena, particularly in patients sensitized to profilins.

Although a substantial proportion of patients exhibited sensitization to food allergens, only approximately 25% had IgE-mediated food allergy (specifically to egg, tree nuts, meat, and milk), confirmed by an oral food provocation test and/or concordance between clear history of adverse reaction to a food and positive sIgE, in agreement with the International Guidelines (18). This distinction is clinically relevant, as food allergy may represent a comorbidity in EoE; however, only foods causing symptoms should be avoided (19).

To our knowledge, this is the first study to investigate panallergen sensitization in a pediatric cohort of EoE patients. Our preliminary findings suggest a possible association between panallergen sensitization and response to PPI therapy in children, as reflected by the higher rate of sensitization in *Group 2* compared with *Group 1*.

In particular, sensitization to profilins and LTPs was negatively associated with PPI response. The high prevalence of LTP sensitization observed in our cohort should also be interpreted in light of the Mediterranean setting, where LTP sensitization is known to be particularly common. One possible explanation is that panallergen sensitization may reflect an earlier and systemic type 2 inflammatory process, which starts with atopic dermatitis (20). In these patients, the esophageal inflammation may be considered the last step of a type-2 atopic march, which may be relatively less dependent on acid-related mechanisms, potentially contributing to a reduced response to PPI therapy.

Previous studies have attempted to identify factors associated with response to PPI therapy, highlighting that older age at diagnosis, male sex, and milder endoscopic findings may be associated with a better response (21).

Notably, patients responsive to PPI tended to be older at symptom onset, had a lower prevalence of atopic dermatitis and showed lower baseline endoscopic severity, as reflected by lower EREFS at diagnosis.

These observations may suggest the presence of two potential clinical phenotypes of pediatric EoE, although this hypothesis should be interpreted cautiously given the limited sample size of the study.

One phenotype may include older patients at diagnosis with no history of atopic dermatitis in early childhood and lower baseline endoscopic severity, showing less panallergen sensitization and better response to PPI therapy. The second phenotype may involve younger patients, with a history of early-onset atopic dermatitis and higher baseline endoscopic severity, fitting more closely with a classic type 2 (Th2) immune activation, with higher panallergen sensitization and lower PPI responsiveness.

Our preliminary findings suggest that patients' sensitization profile, together with clinical characteristics such as early atopic dermatitis, may be associated with treatment response patterns in pediatric EoE.

This study has some limitations, including the small sample size, which limits the statistical power of the study, the lack of a control group and a multivariable analysis. In addition, detailed information on prior treatments, elimination diets, and concomitant allergic therapies was not available for all patients. Moreover, the tertiary referral setting may limit the generalizability of our findings.

These preliminary findings should be interpreted with caution and considered exploratory and hypothesis-generating, requiring confirmation in larger prospective studies.

## Conclusion

Our findings suggest that sensitization to panallergens is common in children with EoE and may be potentially associated with PPI response. However, this hypothesis needs to be further tested in larger, powered studies.

## Data Availability

The raw data supporting the conclusions of this article will be made available by the authors, without undue reservation.

## References

[1] Amil-Dias J Oliva S Papadopoulou A Thomson M Gutiérrez-Junquera C Kalach N. Diagnosis and management of eosinophilic esophagitis in children: an update from the European society for paediatric gastroenterology, hepatology and nutrition (ESPGHAN). J Pediatr Gastroenterol Nutr. (2024) 79(2):394–437. 10.1002/jpn3.12188 38923067

[2] Simon D Cianferoni A Spergel JM Aceves S Holbreich M Venter C. Eosinophilic esophagitis is characterized by a non-IgE-mediated food hypersensitivity. Allergy. (2016) 71(5):611–20. 10.1111/all.12846 26799684

[3] Hill DA Grundmeier RW Ramos M Spergel JM. Eosinophilic esophagitis is a late manifestation of the allergic march. J Allergy Clin Immunol Pract. (2018) 6(5):1528–33. 10.1016/j.jaip.2018.05.010 29954692 PMC6131029

[4] Kliewer K Aceves SS Atkins D Bonis PA Chehade M Collins MH. Efficacy of 1-food and 4-food elimination diets for pediatric eosinophilic esophagitis in a randomized multisite study. Gastroenterology. (2019) 156(Suppl 1):S172–3. 10.1016/s0016-5085(19)37223-3

[5] Mayerhofer C Kavallar AM Aldrian D Lindner AK Müller T Vogel GF. Efficacy of elimination diets in eosinophilic esophagitis: a systematic review and meta-analysis. Clin Gastroenterol Hepatol. (2023) 21(10):2197–210.e3. 10.1016/j.cgh.2023.01.019 36731591

[6] Kagalwalla AF Wechsler JB Amsden K Schwartz S Makhija M Olive A. Efficacy of a 4-food elimination diet for children with eosinophilic esophagitis. Clin Gastroenterol Hepatol. (2017) 15(11):1698–707.e7. 10.1016/j.cgh.2017.05.048 28603055 PMC6448398

[7] Armentia A Martín-Armentia S Martín-Armentia B Santos-Fernández J Álvarez R Madrigal B. Is eosinophilic esophagitis an equivalent of pollen allergic asthma? Analysis of biopsies and therapy guided by component-resolved diagnosis. Allergol Immunopathol. (2018) 46(2):181–9. 10.1016/j.aller.2017.11.001 29338961

[8] Domenech-Witek J Gonzalez-Mendiola R Jover-Cerdá V Pereira-Gonzalez J Guilarte M Luengo O. Description of allergic phenotype in patients with eosinophilic oesophagitis: management protocol proposal. Sci Rep. (2023) 13(1):2226. 10.1038/s41598-023-29602-z 36755125 PMC9906574

[9] Lucendo AJ Gutiérrez-Ramírez L Tejera-Muñoz A Molina-Infante J Arias Á, EUREOS Guidelines Committee. Proton pump inhibitors for inducing and maintaining remission in eosinophilic esophagitis: an updated systematic review and meta-analysis. Clin Gastroenterol Hepatol. (2025) 23(12):2115–27.e21. 10.1016/j.cgh.2025.01.016 40089255

[10] Simon D Straumann A Dahinden C Simon HU. Frequent sensitization to *Candida albicans* and profilins in adult eosinophilic esophagitis. Allergy. (2013) 68(7):945–8. 10.1111/all.12157 23735202

[11] van Rhijn BD van Ree R Versteeg SA Vlieg-Boerstra BJ Sprikkelman AB Terreehorst I. Birch pollen sensitization with cross-reactivity to food allergens predominates in adults with eosinophilic esophagitis. Allergy. (2013) 68(11):1475–81. 10.1111/all.12257 24351068

[12] Armentia A Martín S Barrio J Martín B García JC Vega JM. Value of microarray allergen assay in the management of eosinophilic oesophagitis. Allergol Immunopathol. (2014) 43(5):452–9. 10.1016/j.aller.2014.02.006 24961955

[13] Rossi CM Lenti MV Achilli G Merli S Mauro A Anderloni A. High prevalence of sensitization to non-specific lipid transfer protein in adult patients with primary eosinophilic gastrointestinal disorders in Italy: a single-center series. Clin Mol Allergy. (2022) 20(1):8. 10.1186/s12948-022-00174-z 35858948 PMC9301857

[14] Hogan MB Chawla V Scherr R Allenback G Wonnaparhown A Wilson NW. Aeroallergen, food and panallergen sensitization patterns in eosinophilic esophagitis patients. J Allergy Clin Immunol. (2016) 137(2 Suppl):AB232. 10.1016/j.jaci.2015.12.942

[15] Lucendo AJ Arias Á Redondo-González O González-Cervera J. Seasonal distribution of initial diagnosis and clinical recrudescence of eosinophilic esophagitis: a systematic review and meta-analysis. Allergy. (2015) 70(12):1640–50. 10.1111/all.12767 26392117

[16] Oliva S Arrigo S Bramuzzo M Cisarò F Dabizzi E Di Nardo G. Eosinophilic esophagitis in children and adolescents: a clinical practice guideline. Ital J Pediatr. (2025) 51(1):242. 10.1186/s13052-025-02056-x 40702503 PMC12288368

[17] Barni S Pessina B Scarallo L Renzo S Pieri ES Labriola F. Eosinophilic esophagitis in children: multicenter retrospective study in an Italian cohort. Clin Exp Allergy. (2024) 54(3):225–7. 10.1111/cea.14455 38262705

[18] Santos AF Riggioni C Agache I Akdis CA Akdis M Alvarez-Perea A. EAACI guidelines on the management of IgE-mediated food allergy. Allergy. (2025) 80:14–36. 10.1111/all.16345 39473345 PMC11724237

[19] Arias A González-Cervera J Tenias JM Lucendo AJ. Efficacy of dietary interventions for inducing histologic remission in patients with eosinophilic esophagitis: a systematic review and meta-analysis. Gastroenterology. (2014) 146(7):1639–48. 10.1053/j.gastro.2014.02.006 24534634

[20] O’Shea KM Aceves SS Dellon ES Gupta SK Spergel JM Furuta GT. Pathophysiology of eosinophilic esophagitis. Gastroenterology. (2018) 154(2):333–45. 10.1053/j.gastro.2017.06.065 28757265 PMC5787048

[21] Oliva S Dias JA Rea F Malamisura M Espinheira MC Papadopoulou A. Characterization of eosinophilic esophagitis from the European pediatric eosinophilic esophagitis registry (pEEr) of ESPGHAN. J Pediatr Gastroenterol Nutr. (2022) 75(3):325–33. 10.1097/MPG.0000000000003530 35706095

